# 807. Trimethoprim/Sulfamethoxazole for *Pneumocystis jirovecii* pneumonia prophylaxis in B-cell lymphoma: A multicenter randomized double-blinded placebo-controlled trial

**DOI:** 10.1093/ofid/ofad500.852

**Published:** 2023-11-27

**Authors:** Taksaon Angsutararux, Kiattikon Wongwatcharanon, Wutthiseth Dhitinanmuang, Anothai Chintabanyat, Dusit Jit-Ueakul, Weerapat Owattanapanich, Ekapun Karoopongse, Piyanut Jangkananun, Chavada Piyabun-yanon, Warangkana Munsakul, Methee Chayakulkeeree

**Affiliations:** Pranangklao Hospital, Muang, Nonthaburi, Thailand; Faculty of Medicine Siriraj Hospital, Mahidol University, Bangkoknoi, Krung Thep, Thailand; Ratchaburi Hospital, Muang, Ratchaburi, Thailand; Faculty of Medicine Vajira Hospital, Navamindradhiraj University, Dusit, Krung Thep, Thailand; Faculty of Medicine Vajira Hospital, Navamindradhiraj University, Dusit, Krung Thep, Thailand; Faculty of Medicine Siriraj Hospital, Mahidol University, Bangkoknoi, Krung Thep, Thailand; Faculty of Medicine Siriraj Hospital, Mahidol University, Bangkoknoi, Krung Thep, Thailand; Ratchaburi Hospital, Muang, Ratchaburi, Thailand; Pranangklao Hospital, Muang, Nonthaburi, Thailand; Faculty of Medicine Vajira Hospital, Navamindradhiraj University, Dusit, Krung Thep, Thailand; Faculty of Medicine Siriraj Hospital, Mahidol University, Bangkoknoi, Krung Thep, Thailand

## Abstract

**Background:**

The studies to compare the efficacy and safety of trimethoprim/sulfamethoxazole (TMP/SMX) for *Pneumocystis jirovecii* pneumonia (PJP) prophylaxis in lymphoma patients are scared.

**Methods:**

A multicenter randomized double-blinded placebo-controlled trial was conducted in adult B-cell lymphoma patients receiving R-CHOP. Participants were randomly assigned to received either TMP/SMX (160/800 mg) or placebo trice weekly until 1 month after the last chemotherapy cycle. The primary outcome was PJP at 30 days after cessation of chemotherapy. The secondary outcomes were bacterial infection, survival at day 90 and adverse drug reactions. The modified-intention-to-treat analysis included participants who received at least one dose of the study drugs. Per-protocol analysis included participants who received a complete course of the study drugs or met the study endpoints.

**Results:**

A total of 110 patients were enrolled to the study. Five patients were self-withdrawn after randomization and did not receive any of the study drugs given. Thirteen patients were lost to follow up during the study and 4 patients were prematurely terminated. Therefore, 105 patients were included in the modified intention-to-treat analysis, 55 in TMP/SMX and 50 in placebo group. Eighty-eight patients were included in the per-protocol analysis, 48 in TMP/SMX group and 40 in placebo group. For modified intention-to-treat analysis, 1 patient in the TMP/SMX group developed PJP (1.8%), compared to 0% in the placebo group (*p*-value 1.0). There was indifference of bacterial infection rate between the TMP/SMX and placebo groups (16.3% and 14%, respectively; *p*-value 0.7). The survival rate without PJP events within 90 days was comparable between TMP/SMX and placebo group, 96.4% and 96%, respectively (*p*-value 1.0). Adverse events were significantly higher in the TMP/SMX group compared with the placebo group (18.2% vs. 2%; *p*-value 0.007). Per-protocol analysis also showed no statistically significance in PJP, bacterial infection and 90-day survival between two groups.

**Conclusion:**

TMP/SMX cannot be recommended for primary prophylaxis in lymphoma patients receiving R-CHOP at this time, as it showed higher adverse effects with no benefit on prevention of PJP and bacterial infection as well as survival.

**Disclosures:**

**All Authors**: No reported disclosures

